# General Practitioner Time Availability Per Inhabitant Per Year: A New Indicator to Measure Access to Primary Care

**DOI:** 10.3389/frhs.2022.832116

**Published:** 2022-04-06

**Authors:** Laura Beer, Christine Cohidon, Nicolas Senn

**Affiliations:** Department of Family Medicine, Centre of Primary Care and Public Health (Unisanté), University of Lausanne, Lausanne, Switzerland

**Keywords:** primary care, general practice, access, shortage, availability

## Abstract

**Introduction:**

The number of general practitioners (GPs) per inhabitant, used commonly as an indicator of primary care (PC) access, reports only imprecisely on the true availability of GPs. The aim of this study is to develop a new PC access indicator that better reflects the availability of GPs to take care of patients at the population level, the average GP time availability per inhabitant per year.

**Methods:**

We extracted the data from the Commonwealth Fund International Health Policy Survey of Primary Care Physicians conducted in 2015, including 11 Western countries and 12,049 randomly drawn GPs. We built the indicator by combining two questions about weekly workload in hours and percentage of time spent on face-to-face contact with patients. The indicator was then adjusted regarding the number of GPs, the weeks worked per year and the country's population size.

**Results:**

On average, GPs worked 43 h a week. The average time spent on face-to-face contact with patients was 30.5 h/week (35 h including emails and telephone contact), ranging from 22 h in Sweden and 38 h in France. The mean time available of GPs for face-to-face contact was 69 min/inhabitant/year, ranging from 38 min in Sweden to 118 min in Australia. Including email and telephone contact, the mean contact time rose to 79 min/inhabitant/year, ranging from 48 min in Sweden to 127 min in Australia.

**Conclusion:**

The new indicator provides an accurate and sensitive estimate of a GP's true time availability at the population level. Results should be interpreted in the context of PC workforce organization, which may help guide GP workforce development.

## Introduction

Access to care is a key dimension to assess the performance of health care systems (1). However, access is multidimensional, and so difficult to define and measure with various definitions co-existing in the literature (1–4). For Levesque et al., access is defined as, “the possibility to identify healthcare needs, to seek healthcare services, to reach the healthcare resources, to obtain or use health care services, and to be offered services appropriate to the needs for care (2),” which is a very broad definition, reflecting the intricate aspect of care access.

In most countries, general practitioners (GPs) are the main health providers of primary care (PC) and entry point to the health care system. Many Western countries are concerned about a potential reduction in access to care because of “GP shortage” (5). Indeed, aging GPs (6) and the reduced GP working time reported in many countries could be causes of the perceived decrease in the number of GPs. However, many other factors including work organization (teamwork, inter-professional collaboration) and the increasing needs of the population (multimorbid health problems, demographic changes of aging populations) strongly influence PC access. In addition, entry to PC may be regulated at the system level in different ways, for example, by restricting the choice of provider (referring doctor, gatekeeping) or using financial incentives/barriers for patients. Furthermore, the usual indicator used to judge GP shortage is medical density, which is highly imprecise for guiding health policy. Indeed, medical density does not consider geographical differences within a country, or the number of hours worked per week by GPs, the activities performed at work, or the number of patients seen. In such a complex context with multiple interacting factors, measuring, characterizing and interpreting GP shortage and subsequently its consequence on PC access remains highly speculative and subject to political influences (7, 8).

Several indicators assessing the multiple dimensions of PC access can be found in the literature. For evaluation of the sub-dimension of “availability & accommodation,” some direct indicators based on simple questions concerning the medical density (i.e., number of GP per 100,000 inhabitants) (9–11) or the first contact [for example, ease of a timely appointment with a primary health care provider (12, 13), distance to the GP surgery (10)] are usually proposed. Other, more complex indicators, for example geographical or spatial access indicators (14–16) are described. Furthermore, several indirect indicators including payment modalities, interdisciplinary workforce organization or health equity can be found (17–19).

However, none of these access indicators adequately reflects the time available of the GP to spend with patients. In light of this, we introduce the hypothesis that estimating the time that each inhabitant could theoretically spend with his/her GP is a sensitive and valuable measure of access to PC. The aim of this study, then, is to develop a new indicator measuring the average GP time availability per inhabitant per year at the country level and use it to indicate the variability of this parameter across several countries.

## Methods

### Commonwealth Fund International Surveys

We extracted data from the Commonwealth Fund International Health Policy Survey of Primary Care Physicians conducted in 2015 (20, 21).

The Commonwealth Fund is a private American non-profit foundation devoted to promoting a high performing health care system achieving better access, improved quality, and greater efficiency, with a focus on society's most vulnerable groups. For more than a decade, the Commonwealth Fund has been carrying out health policy surveys that provide international comparisons on health policy topics. In 2015, the Commonwealth Fund conducted an international survey of nationally representative random samples of primary care physicians from 11 countries, including Australia, Canada, France, Germany, New Zealand, the Netherlands, Norway, Sweden, Switzerland, the United Kingdom and the United States. The survey was designed to explore and collect reliable health-related data of primary-care physician practices and experience, including their perception of patient access and their preparedness to manage the care of patients with complex needs and coordinate care for patients with chronic conditions.

### Population and Data Collection

The survey protocol has been published elsewhere (20, 21). In brief, GPs were drawn from government or private lists of primary care practitioners in each country. As the role, level of professional training and functional area of practice of the practitioners responsible for the PC may vary across countries; physician specialties (general practice, family physicians and internists) were determined by country experts. To make the samples comparable across countries, a proportional number of pediatricians were also included in countries in which GPs only take care of adults (US, Germany and Switzerland). A unique questionnaire was revised by experts in each country, adjusted for country-specific wording, and translated as needed to assure comparability across countries. Data were weighted by the Commonwealth Fund for the 2015 International Health Policy Survey of Primary Care Physicians based on known population parameters for each country to ensure that they were representative of the primary care physician population.

### Data

We based our study on two main questions of the survey: “Thinking about your medical practice, estimate how many hours a week you typically work”; “In a typical week, about what percentage of time do you spend on the following: a. Face-to-face contact with patients, b. other contact with patients (e.g., email or telephone) (Note: does not need to add up to 100%).”

GP socio-demographic data completed the set of retained variables.

Other variables investigating practices' organizational characteristics including collaboration with health professionals such as nurses or case managers, the use of electronic patient medical records, the possibility of electronic exchange of a patient's clinical summaries, laboratory or diagnostic test results with doctors outside the practice, and the length of a routine visit were used to discuss and interpret our indicator.

Finally, to obtain the “annual availability time of GPs” we considered the average number of weeks per year that GPs estimated they work each year using data mainly from an internal survey conducted in 2012, the QUALICOPC survey (22). Besides Switzerland, the data concerning the number of GPs per country in 2015 came from the SSRS society in charge of the Methodology Report of the Commonwealth Fund International Health Policy Survey of Primary Care Doctors conducted in 2015 (20). The data relating to the number of GPs in Switzerland in 2015 were from the Swiss Medical Association (FMH). The number of inhabitants per country in 2015 came from OECD (Organization for Economic Co-operation and Development) (see Appendix Exhibit A1).

### Statistical Analysis

We performed all statistical analyses using the 16th version of Stata Software.

We described the sample using descriptive statistics of the GPs' socio-demographic data (age/gender) for each country.

To construct the indicator, we calculated the average number of estimated hours per week the GPs work. Then we multiplied this number by the percentage of time that the GPs count as face-to-face contact with their patients. This allowed us to obtain the average number of hours that the GPs consider they spend on face-to-face contact with their patients each week in each country. Then we extrapolated this number to the yearly available amount of time after deducting the number of weeks of vacation and of continued education. We multiplied the result by the number of GPs in each country and divided the amount of time by the number of inhabitants in each country. Finally, we obtained the theoretical number of minutes that a GP might spend with each inhabitant each year in each specific country.

## Results

Table 1 presents the main characteristics of the sample according to each country. The questionnaire was completed by 12,049 GPs (ranging from 502 in France to 2,905 in Sweden), reflecting a response rate varying from 8% in France to 46.5% in Sweden. The proportion of women differed by 33% in Switzerland to 51.5% in Sweden. The proportion of GPs aged above 54 years ranged from 22% in New Zealand to 49.9% in Switzerland (weighted data).

**Table 1:** Sample demography.

**Medical demography**	**AU**	**CA**	**FR**	**DE**	**NL**	**NZ**	**NO**	**SE**	**CH**	**UK**	**US**	**Total**
Response rate (%)	25.1	31.7	8.1	18.7	40.6	27.7	44.4	46.5	39.0	39.4	30.9	
GP number (N)	747	2,284	502	559	618	503	864	2,905	1,065	1,001	1,001	12,049
Women (%)	37.0	43.9	35.0	44.2	45.2	45.0	40.0	51.5	32.9	49.1	39.9	43.9
<35–44 y	46.9	41.3	36.9	47.1	48.5	46.4	54.3	41.8	34.4	57.6	37.2	44.1
45–64 y	47.6	51.6	60.8	50.2	51.1	52.4	43.6	50.5	62.0	39.7	57.2	50.8
>65 y	5.6	7.1	2.3	2.5	0.4	1.2	2.2	7.7	3.6	2.8	5.7	5.2
Men (%)	63.0	56.1	65.0	55.8	54.8	55.0	60.0	48.5	67.1	50.9	60.1	56.1
<35–44 y	36.1	24.1	16.4	28.4	20.3	31.1	38.8	29.3	11.2	32.2	18.5	25.8
45–64 y	50.5	55.6	70.7	60.8	75.0	62.6	52.4	54.6	70.4	58.2	58.5	59.0
>65 y	13.4	20.3	13.1	10.8	4.7	6.3	8.9	16.1	18.4	9.5	23.1	15.2

*Source: 2015 Commonwealth Fund International Health Policy Survey of Primary Care Physicians*.

*AU, Australia; CA, Canada; CH, Switzerland; DE, Germany; FR, France; GP, general practitioner; NL, The Netherlands; NO, Norway; NZ, New Zealand; SE, Sweden; UK, United Kingdom; US, United States*.

### Available Length of Time of GPs for the Population (Weighted Data) presents the indicator construction for each country

Table 2 presents the indicator construction for each country. In the complete sample, GPs worked an average of 43 h per week, ranging from 36.9 h in New Zealand to 50.4 h in France. The percentage of time spent by GPs on face-to-face contact with their patients varied from 58.1% in Sweden to 82.2% in Australia. The global number of hours that GPs spent per week with their patients (face-to-face, email, telephone) differed by 27.9 h in Sweden to 43.8 h in France. The global average time spent on contact with patients (face-to-face, email and phone contact) was 35 h per week (30.5 h per week face-to-face).

**Table 2:** Indicator construction for each country.

**Country**	**Average GP weekly working hours (weighted)** ***N* = hours**	**% of this time spent on face-to-face contact with patients (weighted)**	**% of this time spent on other than face-to-face contact with patients (weighted)**	**Weekly GP time available for overall contact (weighted)** ***N* = hours**	**Annual GP time for face-to-face contact with patients** ***N* = hours**	**Annual GP time for global contact with patients** ***N* = hours**	**Medical Density per 1,000 population (2015)**	**Annual GP time availability per inhabitant for face-to-face contact** ***N* = minutes**	**Annual GP time availability per inhabitant for global contact** ***N* = minutes**
AU	37.9	82.2	6.5	33.6	1468.5	1579.0	1.340	**118.0**	**127.0**
CA	42.0	76.2	7.9	35.3	1470.5	1624.0	1.130	**99.5**	**110.0**
FR	50.4	76.6	10.7	43.8	1727.5	1969.0	0.675	**70.0**	**80.0**
DE	47.5	69.6	9.8	37.3	1480.0	1699.0	0.541	**48.0**	**55.0**
NL	44.4	62.9	12.9	33.4	1250.0	1504.0	0.569	**42.5**	**51.5**
NZ	36.9	71.4	8.9	29.5	1221.0	1372.5	0.859	**63.0**	**71.0**
NO	41.3	68.6	13.2	33.6	1249.0	1496.0	0.875	**65.5**	**78.5**
SE	37.8	58.1	15.7	27.9	977.0	1241.0	0.644	**38.0**	**48.0**
CH	46.0	69.5	8.4	35.8	1426.0	1595.0	0.958	**82.0**	**91.5**
UK	43.2	69.7	12.6	35.4	1361.5	1612.0	0.926	**75.5**	**89.5**
US	46.9	72.7	11.2	39.0	1590.5	1834.0	0.583	**55.5**	**64.0**

*Sources: 2015 Commonwealth Fund International Health Policy Survey of Primary Care Physicians + see Appendix Exhibit A1*.

*AU, Australia; CA, Canada; CH, Switzerland; DE, Germany; FR, France; GP, general practitioner; NL, The Netherlands; NO, Norway; NZ, New Zealand; SE, Sweden; UK, United Kingdom; US, United States*.

*The bold is used to highlight the indicator*.

The average GP time availability per inhabitant per year (face-to-face contact) was 69 min per inhabitant per year, ranging from 38 min in Sweden, 42.5 min in the Netherlands and 48 min in Germany to 82 min in Switzerland, 99.5 min in Canada and 118 min in Australia. Including email and telephone contact time, the mean time rose to 79 min/inhabitant/year, ranging from 48 min in Sweden to 127 min in Australia. Of note, a country's ranking remains the same after including emails and phone calls.

### Organization Related Variables Which Could Contribute to Discuss the Differences Between Countries

Admitting that health systems and PC's structure and organization may vary across countries, some variables, that investigated the practices organizational characteristics in different domains, have been used to assess and interpret the indicator.

Table 3 describes in detail the work modalities of GPs in each country. Important differences exist in the way GPs organize their time and collaborate with other professionals. Of the countries with a higher average time of GP availability per inhabitant per year (Australia, Canada, Switzerland); conversely, the average consultation length is longer in Switzerland (20 min) than in Australia (15 min) or Canada (17 min). In Switzerland, GPs spend about 20 % of their weekly work hours on administrative tasks compared to 15% in Canada or 10% in Australia. By contrast, only 8% of Swiss GPs in practices collaborate with nurses or case managers compared to 41% in Canada or 75% in Australia. On the other hand, Switzerland and France have the highest percentage of collaboration with other care providers outside of their practices. Access to see “a specialist” seems easier in Switzerland and the Netherlands with <15% of the GPs thinking that their patients have to often wait a long time to see a specialist compared to a range of more than 34% (US) to 70% (Canada) in the other countries. Apart from Canada (69%) and the United States (35%), more than 80% of GPs in practices in the other countries make home visits to their patients. Finally, only 54% of the Swiss GPs use electronic medical records compared to 73% in Canada and 92% in Australia.

**Table 3:** Indicator-influencing variables.

**Country**	**Consultation length (minutes)**	**Collaboration with nurses or case managers within the practice (%)**	**Collaboration with nurses or case managers outside the practice (%)**	**GP electronic medical record use (%)**	**Extent of electronic exchange of clinical patient summaries by GPs (%) < **	**Extent of electronic exchange of laboratory or diagnostic test results by GPs (%)**	**Providers making home visits (%)**	**GPs thinking that patients have to often wait a long time to see a “specialist” (%)**	**Median weekly time for administrative issues (%)**
AU	15.3	75.4	6.2	92.4	39.3	41.1	84.7	57.3	10.0
CA	17.2	40.9	21.5	72.9	20.0	29.4	68.9	70.4	15.0
FR	20.5	12.9	83.3	75.6	49.6	51.9	90.3	64.6	15.0
DE	10.3	20.6	6.8	85.6	22.9	29.1	87.0	62.0	20.0
NL	11.2	83.0	14.9	99.3	79.4	73.2	100.0	11.3	20.0
NZ	15.4	83.0	7.4	99.9	83.6	79.9	95.4	66.0	20.0
NO	19.0	29.1	33.9	99.5	84.4	77.1	98.0	48.4	20.0
SE	23.7	72.6	9.8	99.3	70.5	79.7	94.8	56.4	25.0
CH	19.9	8.4	52.2	54.3	59.6	60.6	88.2	8.9	20.0
UK	10.6	87.4	8.3	98.4	63.9	66.0	99.2	40.9	20.0
US	19.0	42.1	22.8	84.5	44.9	46.9	34.6	34.4	10.0

*Source: 2015 Commonwealth Fund International Health Policy Survey of Primary Care Physicians*.

*AU, Australia; CA, Canada; CH, Switzerland; DE, Germany; FR, France; GP, general practitioner; NL, The Netherlands; NO, Norway; NZ, New Zealand; SE, Sweden; UK, United Kingdom; US, United States*.

## Discussion

The new indicator, average GP time availability per inhabitant per year is statistically easy to calculate once the data has been collected, provides new and meaningful information for assessing workforce availability and access, is sensitive to variations and provides a reliable indicator in comparative studies across countries.

### Meaning of the Indicator

This new indicator provides a more precise estimate than medical density of the time that GPs have available for each citizen and brings some major nuances and potentially interesting advantages. First, by averaging the real time spent with patients, it describes better the true availability of GPs. Second, by providing a time duration instead of number concerning GPs, it has the capacity to capture the comprehensive content of consultations and the allocation of time resources to specific activities and thereby, a possibility to assess the appropriateness of care provided as described by Levesque et al. (2). Indeed, with this indicator data we can distinguish overall time from face-to-face time. This is interesting from a health policy-planning perspective. Last, the number of minutes per capita per year and is probably easier to understand for all health actors even if it remains a global description of the accessibility to PC.

### Comparison Between Countries

In the comparative analysis, GPs from Sweden, the Netherlands, Germany and the United States had the lowest average time available per inhabitant per year for face-to-face contact (38.0–55.5 min). GPs from New Zealand, Norway, France and the United Kingdom had an intermediate average time available per inhabitant per year for face-to-face contact (63.0–75.5 min). GPs from Switzerland, Canada and Australia had the highest average time available per inhabitant per year for face-to-face contact (82.0–118 min). Of note, GP average time available per inhabitant per year for face-to-face contact and overall patient contact (face-to-face, emails and telephone) varied more than 3-fold depending on the country (38 vs. 127 min), even though all eleven countries in the survey are considered industrialized countries.

Compared to the current indicator, such as medical density, the ranking of the countries is similar with the new indicator for some countries such as Australia, Canada, Switzerland, the United Kingdom and New Zealand. However, for other countries, such as Sweden, the indicators differ substantially. Indeed, Sweden has an intermediate medical density (0.644 GP per 1,000 population) but a low average time available per inhabitant per year for face-to-face or overall contact. Along with New Zealand GPs, Swedish GPs work the fewest number of hours per week and spend the highest percentage of their weekly work hours on administrative issues. However, it is important to note that depending on the mode of practice, many administrative tasks (e.g., accounting, medical record keeping, agenda management, establishing prescriptions, medical certificates or medical reports) may be included in the time spent on face-to-face contact. Definitions can also vary across GPs. As with GPs from Switzerland and Norway; GPs from Sweden have a smaller number of weeks of presence in their practices by year (44.5) than the other countries (45.0–47.0). Such differences illustrate that depending on the indicator used, the results concerning “apparent GP availability” for face-to-face contact may diverge greatly across countries. This is relevant as in fine, what matters the most is the time that physicians can spend doing their primary job, taking care of patients, and it may directly affect patient health outcome and satisfaction.

Using indicators that are useful for assessing health care system organization, we noted important differences between countries. However, we should mention that this is only poorly correlated with overall health status of the population. Indeed, Switzerland, Sweden or the Netherlands for example, are considered to have very high-performing health care systems in terms of their population's health status, with longer life expectancy from birth, lower obesity rate and fewer “potential years of life lost” (see Appendix Exhibit A2). However, Switzerland has one of the highest average time available per inhabitant per year for face-to-face contact while Sweden and the Netherlands have much shorter times available for face-to-face contact (see Appendix Exhibit A2). Several hypotheses can explain this apparent paradox. First, it could indicate that the indicator is too specific to modify substantially macroscopic health. Second, it may question the ability of PC to address efficiently key health problems within a given time availability. Finally, it is plausible that a ceiling effect is at play. Indeed, with an overall high quantity of resources that we have in all Western countries (human, financial, etc.), the gain in health might be marginal or random when we add more GPs. This is well-illustrated in a study on quality of access to health care systems (23) published in the Lancet. Indeed, despite similar spending, countries can achieve very different access to care services. For the present indicator, we can thus question the importance to have high GP's time availability in terms of efficiency. But as we will discuss bellow, it is difficult to estimate an “ideal” GP time availability as it depends on different organizational variables.

Furthermore, comparing with other measures of access, we also found that the link between GP time availability and the general assessment of PC accessibility is only partially correlated. For example, in a recent study by the Commonwealth fund (24), countries such as Australia and Switzerland, which have longer GP time availability do not perform especially well in terms of access measured through patient skipping-care rates. On the other hand, countries such as the Netherlands, Sweden or Norway, with low GP time availability show good access levels with limited disparities. These are just some examples that might indicate that other factors, mostly organizational, play important roles in access to PC.

### Factors Influencing GP Time Availability Per Capita

As mentioned above, many factors might influence the time availability of GPs for their patients and for the population. This is the idea behind the new indicator, which may allow a finer time-related measure of a GP's time availability for face-to-face per capita and a better characterization of what GPs do in this time, including primary care organizational activities. In terms of care provided and overall accessibility to health care system, GP time availability per capita captures only a fraction of all problems of access. For example, primary health care systems are uniquely funded and structured and the roles & tasks of GPs differ considerably across countries. One important factor that varies a lot between countries is the consultation's duration. Indeed, for example in the Netherlands, a consultation last 11 min while in Switzerland it is 20 min and 24 in Sweden. In terms of number of patient's contacts per year, we have thus a very different picture with four contacts/year for the Netherlands and Switzerland (although they have a factor-two difference of GP time availability per year) while Sweden has only 1.6 visits per year. On the top of that, we can easily postulate that the content of a 20 min long consultation is very different from an 11 min one. However, few studies have addressed the issue of the content of consultation. It is out of the scope of the present study to explore this aspect, but this new indicator might be useful to investigate the content of care.

Furthermore, other organizational factors at the practice level (consultation structure and collaboration between GPs and other primary care providers) vary from one practice to another. In the present study, Switzerland has one of the highest annual GP availability per capita compared to other countries. On the other hand, GPs collaborate the least with nurses and case managers and are the least likely to use electronic patient medical records (only 54.3% of Swiss GPs are using electronic medical record comparing to 72.9–99.3% for the other countries). Thus, despite higher annual average GP availability, Swiss GPs delegate less tasks to other primary health care providers. This may lead to inferior “effective” time for their patients compared to countries like Sweden with the lowest GP average time available per capita but with comparatively good collaboration with other PC providers including a strong nurse-led gate keeping system (25, 26). Similarly, GPs from the Netherlands have a low average availability per inhabitant per year but almost all collaborate with nurses or case managers within or outside of their practices. Thus, a strong collaboration with other primary health care providers is likely a key element for a “productive” PC system (27, 28) as perhaps GPs with a high annual time availability per capita but who do everything themselves are not able to provide appropriate, effective and necessary care to the population.

In that perspective, it is interesting to note that Kringos et al. (29) reported on the strength of PC in European countries, and that countries ranking the highest for accessibility are not necessarily those with the highest GP time availability. However, the advantage of the new indicator is to be able to guide the GP workforce and improve consultation content by taking into consideration other key organizational factors.

### Estimating the Shortage of GPs

Currently, health policies of several industrialized countries mention a somewhat subjective perception of on-going or future “medical shortage.” However, this notion becomes relative on comparing the different countries of the study with each other. Indeed, as we have seen several factors may explain the differences between the GPs' available time (6, 22, 30–36). In addition, it raises confusion and difficulty of assimilation of two concepts: downsizing and shortage.

For example, in Switzerland the future GP shortage is often voiced by professional associations, the media or in the country's policies (8, 37, 38). This perception is partly based on GP demographic changes (i.e., more than 60% of Swiss GPs were over 54 in 2015), sociological changes of the medical profession (part-time work, feminization) and the tendency for students to pursue specialty training on graduation from medical school. Further, the increasing needs of the population due to aging and multimorbidity may have a role (39). Objectively, several reports acknowledge the growing difficulty of finding a GP or obtaining a prompt appointment (37). Is the solution to bring in more GPs or to transform PC by more inter-professional collaboration? This question is raised rarely, due to strong medical society lobbyism to keep physicians as “lonely players.” In short, we need to be cautious about comparing and interpreting both medical density and GP time availability to conclude a “shortage.” A careful consideration of the overall PC organization is mandatory.

### Limitations and Strengths

There are some limitations to this study. The differences in GP participation rates in the survey (from 8 to 46.5%) may have a repercussion on the collected data. However, the data were weighted to account for differential non-response concerning known geographic and demographic parameters, which may have reduced the selection bias. The mode of data recruitment/completion and incentives varied across the countries. Data were self-reported and were perhaps exposed to declaration biases. The volume of time spent by GPs on face-to face contact or other than face-to face contact with their patients was calculated as a percentage of their estimated weekly working hours. This measuring method is a subjective estimate which may be responsible for a significant bias on the results. Note that in countries where time is charged (as it is the case in Switzerland), this bias is minimized because of census. The number of GPs withheld for each country in our calculation may be imprecise. According to the OECD Health Care Resources Statistics (40), the density of GPs per 1,000 population differs considerably between their sources and those used by the CWF. It is important to note that the density of GPs may also vary according to the particular statistical source due to the definition of PC Physicians. For example, the largest difference in medical density between our study and the OECD concerns the Netherlands, with about 2.7 times more GPs according to the OECD than estimated by our study. Indeed, for the survey, the CWF used a number of 9,641 GPs in 2015; number obtained from NIVEL (Netherlands Institute for Health Services Research). On the other hand, the OECD retained a number of 26,490 GP in 2015. This difference is explained by the definition of the term “GP” used by NIVEL and the OECD. The OECD used the term ≪ Generalist medical practitioners ≫, which includes ≪ General practitioners ≫ and ≪ Other generalist (non-specialist) medical practitioners ≫. This definition is much broader than the one used by NIVEL, which includes only ≪ General practitioners ≫.

A strength of the study is the sample size. A further strength is the standardized methodology of the questionnaire allowing international comparisons. The selected countries are all high-income countries with similar population health output, allowing comparison.

## Conclusion

The new indicator provides an accurate, feasible and sensitive estimate of the true availability of GP time at the population level, which could guide GP workforce development. However, indicator measures need to be interpreted in view of the overall country-level PC organization. Strengthening PC access is complex and requires more than just adding GP time and human resources. Rather, it calls for a multi-disciplinary collaboration, good coordination and continuity of care, clear governance and adequate economic resources. Further work should focus on better characterizing GP time availability per inhabitant per year in terms of content and quality of care. Lastly, this original indicator offers a good opportunity to rethink entirely the roles of GPs regarding both tasks and fulfilling contemporary patient needs.

## Data Availability Statement

The original contributions presented in the study are included in the article/Supplementary Material, further inquiries can be directed to the corresponding author/s.

## Author Contributions

LB: data analysis and interpretation, major contribution in writing the manuscript, and manuscript revision. CC and NS: study initiation and conception, data analysis and interpretation, and manuscript revision. All authors read and approved the final manuscript.

## Funding

This analysis was funded entirely by Unisanté, Department of Family Medicine, University of Lausanne, Switzerland. CWF Health Policy Survey data were obtained free of charge from the Swiss Federal Office of Public Health.

## Conflict of Interest

The authors declare that the research was conducted in the absence of any commercial or financial relationships that could be construed as a potential conflict of interest.

## Publisher's Note

All claims expressed in this article are solely those of the authors and do not necessarily represent those of their affiliated organizations, or those of the publisher, the editors and the reviewers. Any product that may be evaluated in this article, or claim that may be made by its manufacturer, is not guaranteed or endorsed by the publisher.

## Abbreviations

GP: General practitioner
PC: Primary care
AU: Australia
CA: Canada
CH: Switzerland
DE: Germany
NL: The Netherlands
NO: Norway
NZ: New Zealand
SE: Sweden
UK: United Kingdom
US: United States.
