# Supplementary material for: General Practitioner Time Availability Per Inhabitant Per Year: A New Indicator to Measure Access to Primary Care
Source: Front Health Serv. 2022 Apr 6;2:832116. doi: 10.3389/frhs.2022.832116 (PMC10012817; doi:10.3389/frhs.2022.832116)
Supplement: Supplementary file 1 [file Data_Sheet_1.PDF]

Appendix Exhibit A1: External Data used to build the indicator for each country.

Country	Number of weeks per year GPs present in their praxis	Number of GPs in 2015	Number of inhabitants in 2015[15]
**AU**	47.0[1]	31 911[4]	23 815 995
**CA**	46.0[1]	40 275[5]	35 702 908
**FR**	45.0[2]	44 912[6]	66 512 558
**DE**	45.5[1]	44 201[7]	81 686 608
**NL**	45.0[1]	9 641[8]	16 939 925
**NZ**	46.5[1]	3 950[9]	4 595 700
**NO**	44.5[1]	4 544[10]	5 189 898
**SE**	44.5[1]	6 310[11]	9 799 183
**CH**	44.5[1]	7 932[12]	8 282 398
**UK**	45.5[1]	60 290[13]	65 110 034
**US**	47.0[3]	187 100[14]	320 742 673

Sources:

1. 2012 QUALICOPC survey[24]
2. https://drees.solidarites-sante.gouv.fr/etudes-et-statistiques/
3. https://www.medscape.com
4. Australian Government – Dept. of Health – General Practice Statistics 2013-14
5. CMA Masterfile
6. DREES 2013 & 2014
7. National Association of Statutory Health Insurance Physicians Berlin
8. Netherlands Institute for Health Services Research (NIVEL)
9. Medical Council of New Zealand – The New Zealand Medical Workforce in 2012
10. The Registry of GPs at the Norwegian Directorate of Health 2015
11. OneKey database
12. The Swiss Medical Association (FMH) member file, 2015
13. The General Practitioner Register from the General Medical Council, as of December 31, 2013
14. 2014 AMA Physicians Masterfile
15. https://stats.oecd.org/

Appendix Exhibit A2: Health care system profiles

Country	Health expenditure (% of GDP)	Health care spending per capita (US dollars	Out-of-pocket health care spending per capita (US dollars)	Life expectancy at birth (years)	Obesity prevalence (%)	Adults with multiple chronic conditions (2 or more) (%)	Potential years of life lost (years per 100'000 inhabitants)[1]
**AU**	10.3	4791	837	82.6	30.4	15.0	3503
**CA**	11.5	4812	722	82.0	26.3	22.0	4167
**FR**	11.5	4931	463	82.6	17.0	18.0	4067
**DE**	11.5	5848	731	81.1	23.6	17.0	4001
**NL**	10.5	5155	572	81.8	13.4	14.0	3467
**NZ**	9.0	3742	508	81.9	32.2	16.0	-
**NO**	10.5	6064	860	82.7	12.0	16.0	3199
**SE**	10.9	5264	791	82.5	13.1	18.0	3209
**CH**	12.2	7147	2069	83.6	11.3	15.0	2990
**UK**	9.8	3943	629	81.3	28.7	14.0	4186
**US**	8.0	10207	1122	78.6	40.0	28.0	6593

Source: https://www.commonwealthfund.org/international-health-policy-center/countries & https://data.oecd.org/healthstat/potential-years-of-life-lost.htm[1]

Abbreviations: **AU** = Australia; **CA** = Canada; **CH** = Switzerland; **DE** = Germany; **FR** = France; **GDP** = Gross domestic product, **NL** = The Netherlands, **NO** = Norway; **NZ** = New Zealand; **PSHI** = Public Statutory Health Insurance, **SE** = Sweden; **UK** = United Kingdom; **US** = United States.
